# Artificial Intelligence in Pelvic Fracture Diagnosis and Outcome Prediction: A Systematic Review and Meta-analysis

**DOI:** 10.1016/j.mcpdig.2026.100367

**Published:** 2026-04-25

**Authors:** Kevin J. Wang, Aazad Abbas, Geoffrey W. Schemitsch, Matthew Raleigh, Johnathan R. Lex, Hilary Felice, Markku Nousiainen, Cari Whyne, Bheeshma Ravi, Robert Koucheki

**Affiliations:** a Temerty Faculty of Medicine, University of Toronto, Toronto, ON, Canada; b Division of Orthopaedic Surgery, Department of Surgery, University of Toronto, Toronto, ON, Canada; c Institute of Health Policy, Evaluation, and Management, University of Toronto, Toronto, ON, Canada; d Division of Orthopaedic Surgery, Sunnybrook Health Sciences Centre, University of Toronto, Toronto, ON, Canada

## Abstract

**Objectives:**

To synthesize the performance of artificial intelligence (AI) applications for detecting pelvic fractures, classifying severity, and predicting clinical outcomes relative to clinicians.

**Patients and Methods:**

The study was designed as a systematic review and meta-analysis (PROSPERO CRD420251141768). Ovid Embase, Ovid MEDLINE, PubMed, Scopus, and Cochrane CENTRAL were searched for articles published from database inception to September 11, 2025. Studies were included if they evaluated AI models for pelvic ring fractures in adults using pelvic radiographs. Case series, reviews, and abstracts without full data were excluded. Summary level data were independently extracted using a standardized template. Diagnostic metrics were pooled using a random-effects model. Outcomes included pooled sensitivity, specificity, area under the receiver operating characteristic curve (AUC), and accuracy.

**Results:**

Fourteen studies were included. Thirteen studies evaluated radiographic fracture detection or classification (n=31,166 radiographs) and one study evaluated outcome prediction. AI demonstrated high pooled performance: accuracy 0.96 (95% CI, 0.91-0.98; *I*^*2*^=93.3%), AUC 0.94 (95% CI, 0.89-0.97; *I*^*2*^=97.9%), sensitivity 0.90 (95% CI, 0.84-0.94; *τ*^2^=0.42), and specificity 0.93 (95% CI, 0.85-0.97; *τ*^2^=1.30). In 3 studies directly comparing AI with clinicians, AI models showed comparable or marginally superior performance. One study on clinical outcomes reported strong performance for predicting hemodynamic instability (AUC 0.92) and mortality (AUC 0.90).

**Conclusion:**

AI algorithms show promise as supportive tools for pelvic fracture detection, achieving diagnostic performance comparable to expert clinicians. However, included studies exhibit substantial heterogeneity, selection bias, and limited external validation. Large-scale, prospective validation is necessary before widespread clinical adoption.

Pelvic ring fractures are an important cause of morbidity and mortality. Globally, an estimated 4.5 million cases occur annually, amounting to over 2 million years lived with disability.^1^ These injuries can lead to life-threatening complications and long-term disability, and are an important cause of death in older adults.^2^ Anteroposterior pelvic radiographs are the first-line diagnostic tool for high-energy trauma and falls, but roughly 20% of pelvic fractures are initially missed on plain film.^3^ In particular, the posterior pelvic ring can be difficult to visualize because of overlapping pelvic anatomy, contributing to high rates of missed injuries. Studies indicate that up to 94% of the sacrum may be obscured on plain radiographs, with diagnostic sensitivities as low as 20% for sacral fractures and 17% for ischial fractures.^4^^,^^5^ These diagnostic limitations can delay fracture diagnosis and contribute to severe functional decline associated with immobility.

Artificial intelligence (AI), particularly deep learning and computer vision, has emerged as a promising tool to improve imaging in trauma and musculoskeletal care.^6^^,^^7^ Convolutional neural networks have demonstrated high accuracy in fracture detection and have been shown to support clinical workflows in acute trauma settings.^8^^,^^9^ A recent meta-analysis reported that AI achieves pooled sensitivity and specificity of approximately 92%, comparable to clinician performance.^6^ These compelling results have spurred policy changes, including the decision by the United Kingdom’s National Institute for Health and Care Excellence to endorse AI-based fracture detection tools.^10^ Beyond raw detection, AI models have also been developed for prognostic and management applications in orthopedics, using clinical data to predict hemodynamic instability, mortality, and functional outcomes.^7^^,^^11^^,^^12^

Despite these advances, no prior systematic review has specifically addressed AI for pelvic fracture imaging and management. Existing reviews have covered general fractures^6^^,^^10^ and hip fractures,^7^ but the unique aspects of pelvic trauma remain unexamined. To address this, we conducted a systematic review to synthesize the current state of AI applications using plain radiographs for pelvic fractures. Our objectives were to evaluate AI performance in diagnosing pelvic fractures, classifying fracture severity, and predicting clinical outcomes, while comparing these results with the performance of clinicians. We aim to clarify the current capabilities and limitations of these computational tools for one of the most diagnostically challenging tasks.

## Patients And Methods

This study followed the 2020 Preferred Reporting Items for Systematic Review and Meta-Analysis (PRISMA) guidelines.^13^ The study was prospectively registered on PROSPERO (CRD420251141768).

### Search Strategy

A systematic search was conducted across 5 databases (Embase via Ovid, MEDLINE via Ovid, PubMed, Scopus, and Cochrane CENTRAL). The search includes all English-language articles indexed from database inception to September 11, 2025. The following search terms were used in each database:

(1) pelvis OR pelvic OR sacrum OR sacral OR pubic OR ischium OR ilium OR “pelvic ring”
(2) fracture∗ OR broken OR break∗ OR crack∗ or comminut∗ or displace∗
(3) “artificial intelligence” OR AI OR “machine learning” OR ML OR “computer vision” OR “neural network” OR “deep learning” OR DL
(4) 1 AND 2 AND 3

### Eligibility and Article Selection

All randomized controlled trials, cohort studies, prospective and retrospective studies, and case-control studies were considered, whereas editorials, reviews, case series with less than 10 patients, and abstracts without full data were excluded. Studies were limited to adults older than 18 years with suspected or confirmed pelvic ring fractures, including pubic rami, sacral, iliac wing, and sacroiliac joint injuries. Studies diagnosing pelvic fractures from medical imaging were only included if models were based on anteroposterior, inlet, or outlet pelvic radiographs. Studies must have also used one of the following ground truth methods for diagnosis confirmation: pelvis computed tomography (CT), magnetic resonance imaging, or image review with consultant radiologist consensus. Cohorts with acetabular fractures, proximal femoral fractures, and mixed injuries were excluded unless data for pelvic ring fractures were extractable as a distinct subgroup.

Abstracts and full-text articles were screened by a single reviewer (K.J.W.) against the predefined eligibility criteria. Any records where eligibility was uncertain were resolved through the consultation with 2 independent reviewers (A.A., R.K.).

### Data Extraction and Risk of Bias Assessment

Extracted summary level data included algorithm training parameters, area under the receiver operating characteristic curve (AUC), accuracy, sensitivity, and specificity. If studies compared diagnostic performance of clinicians with AI models, we extracted data exclusively from senior specialists (radiologists and orthopedic surgeons), excluding resident trainees. Where multiple experts were reported, we calculated a mean score to minimize the impact of interobserver variability. When studies trained multiple models for the same outcome, the data from the best performing model were recorded, reflecting the likely candidate for clinical use.

The Quality Assessment of Diagnostic Accuracy Studies-2 was used to assess the risk of bias in each of the studies.^14^^,^^15^ This instrument evaluates methodological rigor across 4 domains: patient selection, index test, reference standard, and flow and timing. Each domain was assessed in terms of risk of bias and concerns regarding applicability, and assigned a classification of low, high, or unclear based on the responses to signaling questions. The overall classification of each study was based on the rating of its worst domain. Risk of bias was assessed for all studies by a single reviewer, and uncertainties were resolved through consultation with 2 independent reviewers. Robvis was used to visualize risk of bias assessments.^16^ Publication bias was assessed for the AUC outcome using funnel plots and Egger’s linear regression test, with *P*<.10 defining significant asymmetry.^17^

### Statistical Analyses

Statistical analysis followed the Cochrane Handbook for Systematic Reviews of Diagnostic Test Accuracy.^18^ All statistical analyses were conducted in R (version 4.5.2, 2025, R Foundation for Statistical Computing)^19^ using the meta^20^ and mada^21^ packages. For the meta-analysis of sensitivity and specificity, a bivariate random-effects model (Reitsma model^22^) was used to account for the inherent negative correlation between sensitivity and specificity. Pooled estimates were calculated with 95% CIs. These results were visualized in receiver operating characteristic space with a 95% confidence ellipse generated around the pooled mean to illustrate the uncertainty of the summary estimate. Quantitative syntheses of the AUC and accuracy were performed using a generic inverse-variance random-effects model on the logit scale. When CIs were unavailable, variances were estimated using the Hanley-McNeil method^23^ for AUC and binomial distribution for accuracy. For the assessment of publication bias, the Hanley-McNeil method was applied to calculate the standard error for all included studies. This approach was chosen to maintain a uniform variance structure across the funnel plot and Egger linear regression test. Heterogeneity was characterized by the between-study variance (*τ*^2^) from the bivariate model and by the *I*^*2*^ statistic for AUC and accuracy.

## Results

### Study Selection and Characteristics

**Figure 1:**
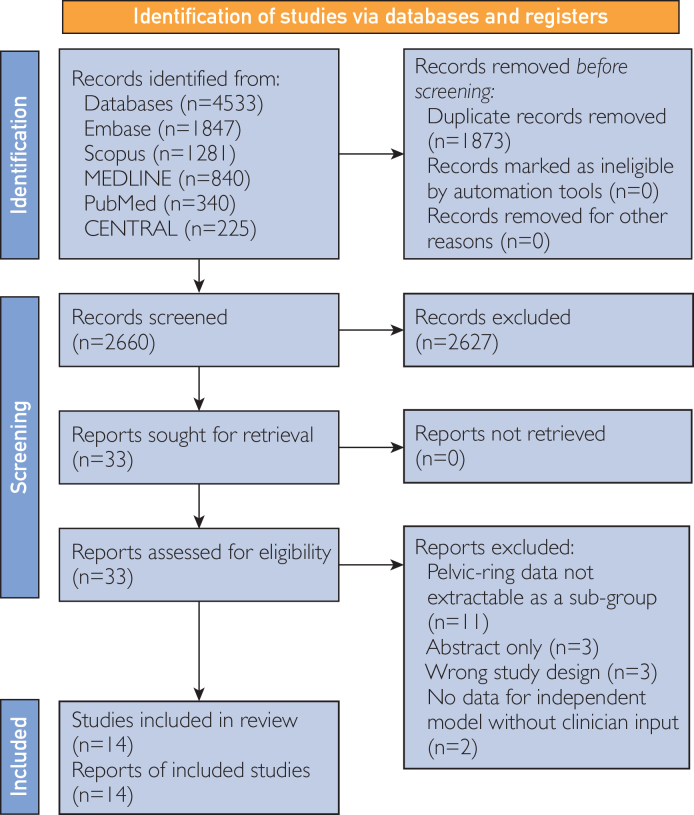
Preferred Reporting Items for Systematic Review and Meta-Analysis (PRISMA) 2020 flow diagram of the study selection process.

The search yielded 4,533 citations, of which 14 studies ultimately met inclusion criteria (Figure 1). Publication dates ranged from 2020 to 2025. The characteristics of included studies focusing on fracture diagnosis and classification (n=13) are summarized in Table 1. Thirteen studies evaluated AI models for diagnostic imaging tasks,^24^, ^25^, ^26^, ^27^, ^28^, ^29^, ^30^, ^31^, ^32^, ^33^, ^34^, ^35^, ^36^ and one study used AI models to predict hemodynamic instability and mortality in patients with closed pelvic fractures.^37^ Two studies trained models to classify fractures according to the Association for Osteosynthesis and Orthopedic Trauma Association fracture classifications.^30^^,^^31^ A cumulative total of 31,166 pelvic radiographs were used across the training, validation, and testing of these models. Eleven studies derived radiographs retrospectively from medical records,^24^, ^25^, ^26^^,^^29^, ^30^, ^31^, ^32^^,^^34^, ^35^, ^36^, ^37^ one study prospectively enrolled patients,^33^ and two studies^27^^,^^28^ used the same prelabeled public dataset of images.^38^

**Table 1:** Characteristics of Included Studies Evaluating Artificial Intelligence for the Diagnosis and Classification of Pelvic Fractures

First author, y (country)	Input imaging	Output type	Best performing algorithm	Output	Pelvic X-rays (n)	Training size	Validation size	Testing size	Ground truth
Cheng,^24^ 2021 (Taiwan)	PXR	Multi (3)	PelviXNet (DenseNets)	Fractured (pelvic area); fractured (hip); nonfractured	5204	100%	NR	36.3%	Image review by group of clinicians
Chen,^25^ 2020 (USA)	PXR, various views	Binary	DenseNet-121	Fractured (anterior pelvis); nonfractured	2359	70%	10%	20%	Labeled by experienced clinicians
Inagaki,^26^ 2022 (Japan)	AP PXR cropped, sacroiliac joint and sacrum	Binary	InceptionV3	Fractured; nonfractured	2238	91%	NR	9%	Labeled by 2 orthopedic surgeons based on CT
Islam,^27^ 2025 (Bangladesh)	PXR	Binary	MobileNet	Fractured; nonfractured	876	72%	18%	10%	Labels derived from a public database
Kassem,^28^ 2023 (Egypt)	PXR	Binary	GoogleNet	Fractured; nonfractured	876	70%	15%	15%	Labels derived from a public database
Kitamura,^29^ 2020 (USA)	PXR, frontal, oblique, inlet, outlet	Multi (5)	DenseNet-121	Nonfractured; pelvic ring; proximal femur; acetabular; complex	7337	70%	NR	30%	Labels derived from established radiology archive; image review by an MSK radiologist
		Binary		Fractured (pelvic ring); nonfractured					
Lee,^30^ 2024 (South Korea)	AP PXR	Multi (4)	Inception-ResNet-V2	AO/OTA type A, type B, and type C; nonfractured	940	NR	NR	100%	Radiologist report; CT; each image reviewed by a trauma surgeon
		Binary		Fractured; nonfractured					
Park,^31^ 2024 (South Korea)	AP PXR	Multi (4)	Radiomics-based approach	AO/OTA type A, type B, and type C; nonfractured	1169	70%	NR	30%	Radiologist report; orthopedic specialist’s opinion; CT scans; evaluated by a trauma surgeon with >10 years of experience
Rahman,^32^ 2024 (Japan)	PXR and synthesized PXR from 3D-CT	Binary	ResNet101	Fractured; nonfractured	7244	NR	NR	NR	Consensus by an expert radiologist and doctors
Rosa,^33^ 2023 (Italy)	PXR	Binary	DCNN via Detectron2	Fractured; nonfractured	235	NR	NR	100%	Senior radiologist with >10 years of experience; CT
Ruitenbeek,^34^ 2025 (Netherlands)	PXR, AP, lateral, oblique	Binary	DCNN	Fractured (pelvic); nonfractured	1008	NR	NR	100%	Radiology resident and staff MSK radiologist
Tsai,^35^ 2025 (Taiwan)	PXR	Multi (4)	DenseNet-121	Iliac wing and ischial spine fracture; iliac wing fracture ischial spine fracture; localized fracture (neither iliac wing nor ischial spine)	100	70%	15%	15%	Two board-certified radiologists
Wu,^36^ 2021 (China)	PXR	Binary	ResNext101	Fractured (pelvic); nonfractured	1580	NR	NR	20%	Labeled by 2 radiologists; reviewed by the chief physician

Abbreviations: 3D-CT, 3-dimensional computed tomography; AO/OTA, Association for Osteosynthesis and Orthopedic Trauma Association; AP, anteroposterior; DCNN, deep convolutional neural network; MSK, musculoskeletal; NR, not reported; PXR, pelvic X-ray.

### Performance of Artificial Intelligence in the Diagnosis of Pelvic Fracture

While most studies (n=12) developed convolutional neural networks for fracture diagnosis,^24^, ^25^, ^26^, ^27^, ^28^, ^29^, ^30^^,^^32^, ^33^, ^34^, ^35^, ^36^ one study used a radiomics-based approach.^31^ Performance was most frequently assessed using AUC (n=9),^25^, ^26^, ^27^^,^^29^, ^30^, ^31^, ^32^^,^^34^^,^^36^ accuracy (n=7),^24^^,^^26^, ^27^, ^28^^,^^30^^,^^33^^,^^35^ and sensitivity and specificity (n=7).^24^^,^^26^^,^^28^^,^^30^^,^^33^^,^^34^^,^^36^ Three studies additionally provided a direct benchmark comparison against expert clinicians on identical test sets (Table 2).^24^^,^^26^^,^^33^ Two studies were excluded from the quantitative synthesis: Park et al^31^ due to the absence of binary AUC data and Tsai et al^35^ for reporting accuracy limited to specific anatomical regions (iliac wing or ischial spine) rather than overall pelvic fractures.

**Table 2:** Diagnostic Performance of Artificial Intelligence Models and Clinicians for Pelvic Fracture Detectiona,b,^c^

First author, y	Best performing algorithm	ML model					Clinician		
		AUC (95% CI) ± SD	Accuracy (95% CI) ± SD	Sens.	Spec.	F1-score	Accuracy	Sens.	Spec.
Cheng,^24^ 2021	PelviXNet (DenseNets)	–	0.945	0.92	0.97	–	0.94	0.885	0.995
Chen,^25^ 2020	DenseNet-121	0.9771	–	–	–	–	–	–	–
Inagaki,^26^ 2022	InceptionV3	0.989 (0.975-1)	0.935	0.88	0.99	0.931	0.5525	0.5575	0.5475
Islam,^27^ 2025	MobileNet	0.991	0.9943	0.9943	–	0.9943	–	–	–
Kassem,^28^ 2023	GoogleNet	–	0.985	0.9701	1	–	–	–	–
Kitamura,^29^ 2020	DenseNet-121	Multiclass: 0.81 (0.78-0.84) Binary: 0.86 (0.85-0.87)	–	–	–	–	–	–	–
Lee,^30^ 2024	Inception-ResNet-V2	Overall: 0.85 Binary: 0.878	Overall: 0.79 Binary: 0.919	Overall: 0.644 Binary: 0.959	– Binary: 0.736	Overall: 0.624 Binary: 0.951	–	–	–
Park,^31^ 2024	Radiomics-based method	Overall: 0.75 ± 0.06	–	–	–	–	–	–	–
Rahman,^32^ 2024	ResNet101	0.9327	–	–	–	0.895	–	–	–
Rosa,^33^ 2023	DCNN via Detectron2	–	0.89 (0.84-0.93)	0.7674	0.9167	–	0.9362	0.7907	0.9688
Ruitenbeek,^34^ 2025	DCNN	0.91 (0.88-0.94)	–	0.867	0.802	–	–	–	–
Tsai,^35^ 2025	DenseNet-121	–	Iliac wing: 0.921 ± 0.014 Ischial spine: 0.940 ± 0.012	Iliac: 0.891 Ischial: 0.887	–	Iliac: 0.882 Ischial: 0.904	–	–	–
Wu,^36^ 2021	ResNext101	0.925	–	0.8530	0.9430	–	–	–	–

^b^For studies that reported statistics for multiple validation folds and/or clinicians, the mean values were calculated.

^c^Multiclass refers to the AUC value for pelvic ring fracture detection in the multiclass output model. Binary refers to the AUC value in the binary output model. Overall refers to the mean AUC value across all possible output classification combinations.

a Abbreviations: AUC, area under the receiver operating characteristic curve; DCNN, deep convolutional neural network; ML, machine learning; Sens., sensitivity; Spec., specificity.

b For studies that reported statistics for multiple validation folds and/or clinicians, the mean values were calculated.

c Multiclass refers to the AUC value for pelvic ring fracture detection in the multiclass output model. Binary refers to the AUC value in the binary output model. Overall refers to the mean AUC value across all possible output classification combinations.

**Figure 2:**
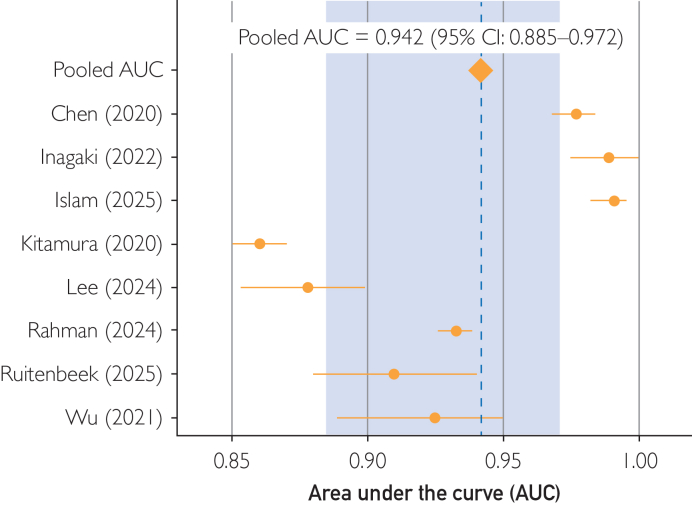
Pooled area under the curve (AUC) for artificial intelligence models in detecting pelvic fractures.

**Figure 3:**
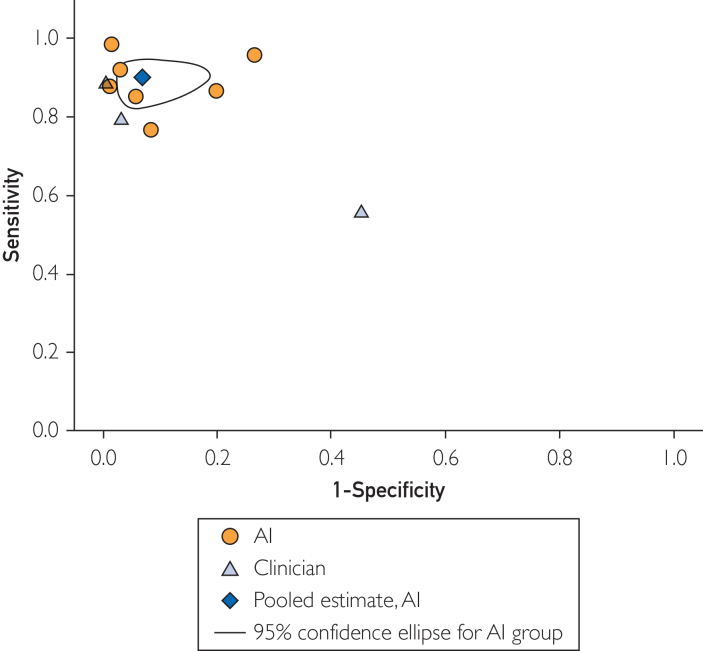
Sensitivity and specificity of artificial intelligence (AI) models and clinicians in diagnosing pelvic fractures on pelvic radiographs.

AI models demonstrated high overall performance, with a pooled accuracy of 0.960 (95% CI, 0.907-0.983; *I*^*2*^=93.3%) and an AUC of 0.942 (95% CI, 0.885-0.972; *I*^*2*^=97.9%) (Figure 2). In terms of operating characteristics, pooled sensitivity was 0.900 (95% CI, 0.841-0.939; *τ*^2^=0.416) and specificity was 0.932 (95% CI, 0.845-0.971; *τ*^2^=1.299). Clinician performance varied widely across the 3 studies, largely due to a single study with performance ranging from 0.55 to 0.56 (Table 2). The pooled AI estimate slightly outperformed all clinician cohorts in sensitivity but underperformed 2 clinician groups in specificity (Figure 3). In direct comparison, 2 studies found that their AI model was marginally superior to their physician comparison across all metrics (accuracy, sensitivity, and specificity),^24^^,^^26^ whereas 1 study reported slightly inferior AI performance^33^ (Table 2).

### Performance of Artificial Intelligence in Predicting Outcomes

Wang et al^37^ developed binary AI models to predict acute hemodynamic instability and in-hospital mortality using a retrospective dataset of 133 patients with closed pelvic fracture. The cohort was split into a training set (n=115) and a testing set (n=18). The study constructed the hemodynamic instability model using 11 clinical features as inputs, categorized into vital signs (heart rate and systolic and diastolic blood pressure), laboratory markers (white blood cell count, fibrinogen, creatinine, pH, *p*CO_*2*_, *p*O_*2*_, and lactic acid), and injury characteristics (Tile classification). The mortality model used 4 established trauma indices: the Injury Severity Score, Glasgow Coma Scale, Trauma and Injury Severity Score, and Acute Physiology and Chronic Health Evaluation II. Among the 7 algorithms evaluated, the random forest model demonstrated the best performance for both outcomes, with an AUC of 0.92 (accuracy 0.86) for hemodynamic instability and 0.90 (accuracy 0.91) for mortality.

### Risk of Bias Assessment

The overall risk of bias across the included literature was moderate to high, as summarized in Supplemental Figure 1 (available online at https://www.mcpdigitalhealth.org). Of 14 studies, 13 (93%) were judged to have unclear or high risk of bias. The principal sources of potential bias were due to concerns about patient selection and unclear decision thresholds. Most studies used retrospective, nonconsecutive sampling or did not clearly report sampling methods, raising the possibility of spectrum bias and the exclusion of difficult-to-diagnose cases. Although AI models mathematically produce results without human interference, most studies were rated as unclear or high risk for their index test because of a lack of clarity surrounding the decision thresholds for what is deemed to be a fracture. The reference standard was judged to be low risk, as most studies used robust ground truths such as CT scans or consensus of expert physicians. The flow and timing were generally low risk, as most studies used a cross-sectional design. Individual study assessments are detailed in Supplemental Figure 2 (available online at https://www.mcpdigitalhealth.org).

Visual inspection of the funnel plot for AUC outcomes suggests asymmetry and small-study effects (Supplemental Figure 3, available online at https://www.mcpdigitalhealth.org). However, this asymmetry may be an artifact of the logit transformation and the mathematical ceiling effect of AUC, which can disproportionately inflate the logit standard error for studies reporting near-perfect AUC. The Egger test did not indicate significant small-study effects (*P*=.22). Because of the limited number of studies included in the meta-analysis (n=8), the results of the Egger test should be interpreted with caution, and the possibility of publication bias cannot be ruled out.

## Discussion

This review demonstrated that deep learning models have very high AUC, accuracy, sensitivity, and specificity (>0.9), indicating their excellent overall performance in identifying pelvic fractures. The pooled AI performance was comparable to that of physicians in 2 studies and superior in 1 study. We also identified one study on clinical outcome prediction, which developed a capable random forest model using physiologic and injury data to predict acute hemodynamic instability and in-hospital mortality.^37^ This suggests that AI may also help risk-stratify patients with pelvic trauma, although the evidence is preliminary.

Our pooled findings are consistent with prior studies of AI in general fracture detection. Kuo et al,^39^ for instance, reported that the pooled sensitivity and specificity for both AI models and clinicians were near parity, ranging from approximately 91% to 92%. A separate meta-analysis of 100 studies showed an AI sensitivity of 91.4%, a specificity of 92.1%, and an AUC of 0.968.^6^ In addition, Lex et al^7^ analyzed models specific to hip fractures and reported a pooled sensitivity of 89.3% and a specificity of 87.5%. Thus, AI models for pelvic fracture detection appear at least as accurate as those for general or hip fractures, although the body of AI research specific to the pelvis remains limited.

Given the similar performance between AI and expert clinicians, the most practical application of AI may be its role as a concurrent aid in the trauma bay, especially during the “golden hour” of resuscitation. Early detection of pelvic ring fractures associated with hemorrhage and mortality can activate time-sensitive treatments, such as early pelvic binder placement, transfusion protocols, expedited CT, and earlier mobilization of trauma and orthopedic resources. Pelvic radiographs are often first interpreted by emergency physicians and trauma team leaders under time pressure, including overnight when formal radiology reporting may be delayed until the next morning.^40^ A missed fracture during this early window can be consequential, as each additional hour of delay to surgery increases the odds of systemic complications by 0.4%.^41^ The implementation of an AI visualization overlay that flags suspicious areas could reduce diagnostic errors and expedite diagnoses when expert radiologists and orthopedic surgeons are unavailable. As Lindsey et al^8^ demonstrated in wrist imaging, a deep learning model enhanced emergency physicians’ clinical judgment and reduced their false-negative rate by 47%. This type of benefit may be more pronounced in pelvic imaging, given the greater complexity of pelvic anatomy.

An additional opportunity for clinical translation is improving detection and triage of fragility pelvic fractures in older adults, an increasingly common source of immobility and morbidity.^42^^,^^43^ Insufficiency fractures frequently occur after low-energy trauma and may be radiographically subtle, with pain and immobility as primary presentations.^44^ AI applications could potentially reduce false negatives on initial radiographs and support clinicians’ decision to order confirmatory cross-sectional imaging, leading to earlier diagnoses. However, this type of application relies on robust validation in geriatric and osteoporotic cohorts, where imaging quality, positioning, degenerative change, and prior implants may differ substantially from younger trauma populations and could introduce domain shift.

A major limitation of the evidence base is the reliance on retrospective case-control studies. Most algorithms were trained on datasets with high fracture prevalence, in some cases as high as 83% of all images (Supplemental Table 1, available online at https://www.mcpdigitalhealth.org). In comparison, the real-world prevalence of pelvic fracture is estimated to be 6%,^45^ decreasing the positive predictive value and utility of AI models in clinical practice. However, having balanced groups typically strengthens AI model performance, reducing model bias of predicting the more common event. Indeed, other meta-analyses have similarly found pervasive bias risk in AI radiology research.^39^ In addition, the pooled estimates showed substantial heterogeneity (*I*^*2*^>90% for accuracy and AUC), reflecting variability in study design, patient cohorts, and technical approach. Most studies used a single-center design and did not validate their models on independent external datasets, limiting generalizability. Ground truth methods differed between studies, with some relying on radiologist consensus on X-rays and labels generated by unspecified third parties. Finally, many AI models were evaluated only with small test sets, constraining confidence in the pooled metrics. Although our meta-analysis highlights strong average performance, these results should be interpreted with caution given the limited literature, variability, and methodological limitations of the underlying studies.

Further research is needed to measure the safety and performance of AI tools before they can be widely adopted. Ruitenbeek et al’s^34^ study was the only included study to perform external validation, testing an AI model trained on Indian data in the Dutch population. However, its retrospective design opens the possibility of spectrum bias, as previously discussed. Prospective, multicenter validation studies are essential to confirm performance in diverse clinical settings and accurately gauge positive predictive value. Future studies that integrate AI into the workflow of emergency departments and directly measure its impact on decision-making and patient outcomes are necessary. Model explainability is another priority, and future algorithms should provide localization through salience maps or bounding boxes. Requiring physicians to evaluate AI-highlighted regions, agree or disagree with suggestions, and integrate the information into a broader clinical assessment could keep clinicians involved in the process and preserve their autonomy. From a regulatory standpoint, this augmented decision-making framework helps mitigate fears of over-reliance, loss of skill, or unclear algorithmic decision pathways. Professional societies and guideline bodies are beginning to weigh in,^10^ but further pelvic fracture validation is needed to guide specific recommendations.

To our knowledge, this study is the first comprehensive synthesis of AI algorithms developed specifically for identifying pelvic fractures. Conducting a review in this emerging field came with several challenges and limitations. First, the small number of eligible studies limited our ability to compare model architectures or performance differences across algorithm types. In addition, we were unable to provide a summary receiver operating curve because several studies did not provide the necessary data, such as contingency tables or the number of true-positive and true-negative radiographs.

## Conclusion

In summary, AI shows promise as an adjunct for pelvic fracture detection on radiographs, with pooled performance metrics suggesting that it can match clinician experts. These findings contribute to the growing literature on AI to aid the complex diagnosis of pelvic fractures. However, the high pooled accuracy and AUC should be interpreted with caution due to limited study external validation and bias. Future research should perform cross-validation with diverse populations and use algorithms prospectively in clinical settings to directly measure its true clinical value.

## Potential Competing Interests

The authors report no competing interests.

## Ethics Statement

Given that this study was conducted as a systematic review and meta-analysis, there was no need for institutional review board approval or informed consent from participants.

## Data Sharing Statement

The data used in this systematic review are available in the publication and appendix and can be requested from the corresponding author.

## Supplemental Online Material

Supplemental Material

Supplemental Checklist
