# Supplementary material for: Artificial Intelligence in Pelvic Fracture Diagnosis and Outcome Prediction: A Systematic Review and Meta-analysis
Source: Mayo Clin Proc Digit Health. 2026 Apr 25;4(2):100367. doi: 10.1016/j.mcpdig.2026.100367 (PMC13241727; doi:10.1016/j.mcpdig.2026.100367)
Supplement: Supplemental Material [file mmc1.docx]

**Supplemental Material**


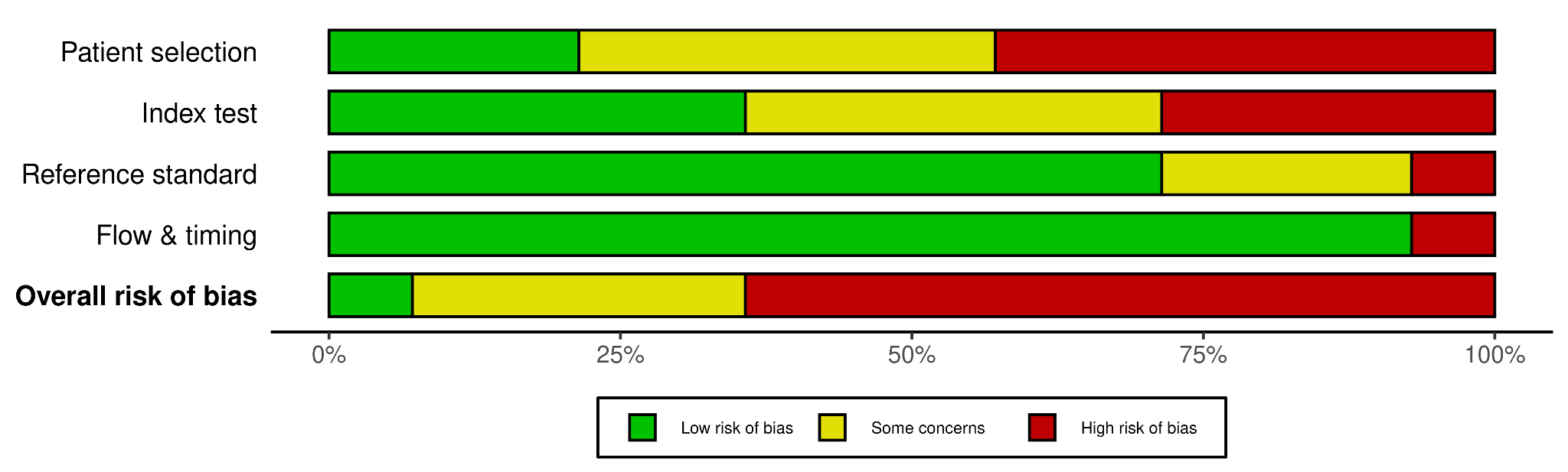


**Supplemental Figure 1. Aggregate risk of bias assessment.**

Percentage of studies rated as low, high, or unclear risk across QUADAS-2 domains.


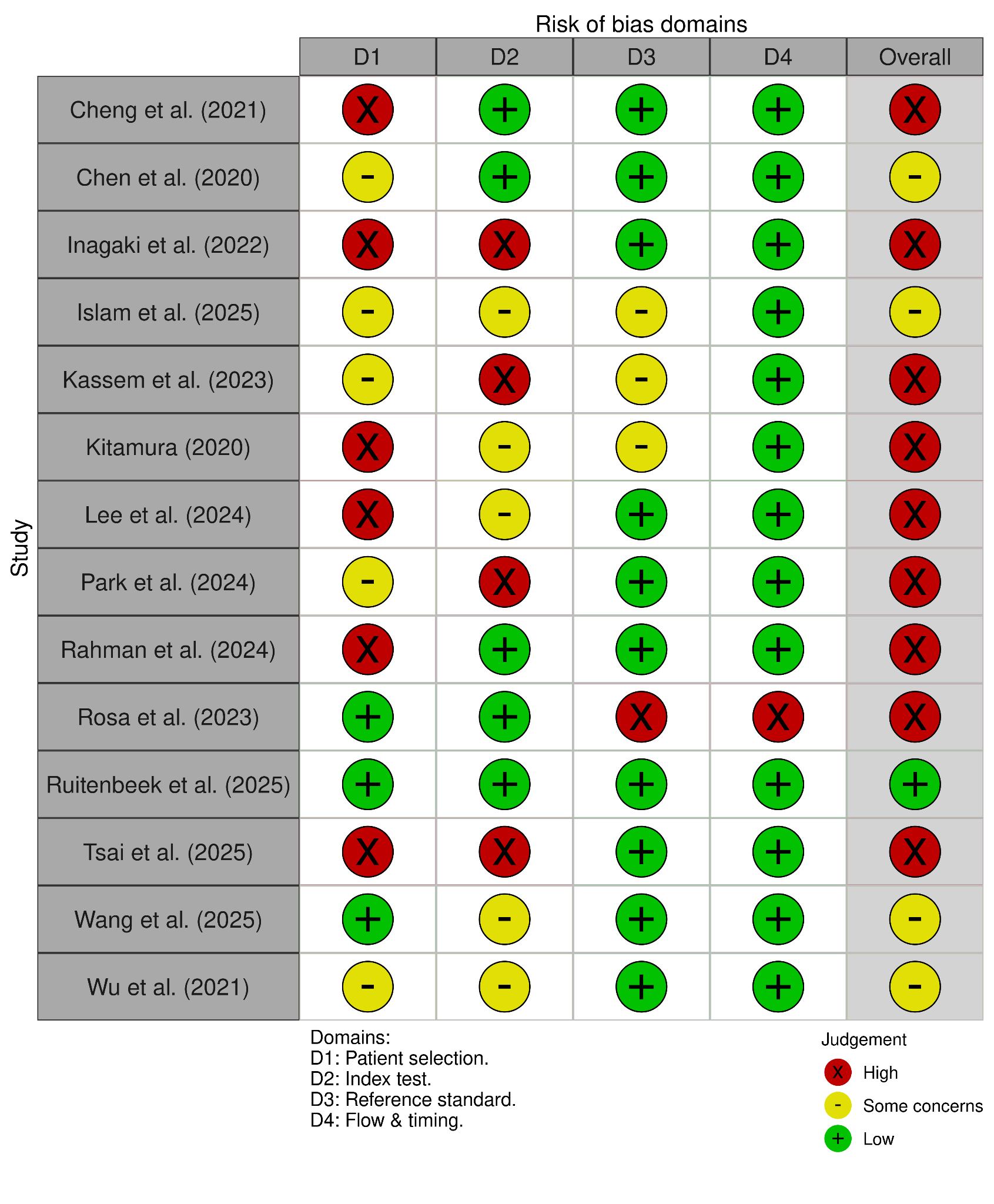


**Supplemental Figure 2. Risk of Bias Summary.**

Review authors' judgments about each risk of bias domain for each included study.


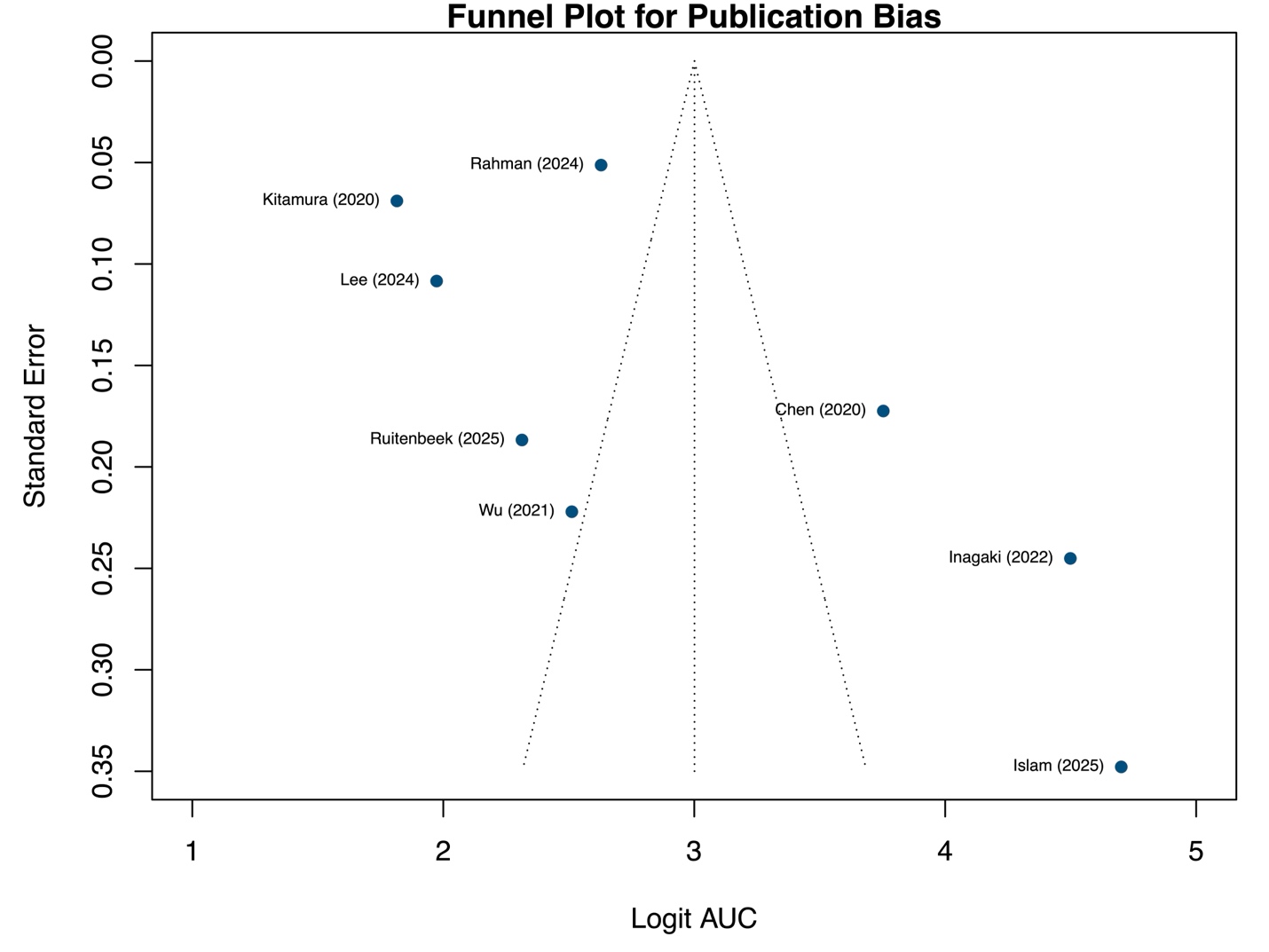


**Supplemental Figure 3. Funnel Plot for Area Under the Curve (AUC) Outcomes.**

**Supplemental Table 1. Summary of Radiographic Datasets and Model Validation Strategies.**

| **First Author, Year** | **Number of X-rays Used** | **Images (n) positive for pelvic fracture** | **Images (n) negative for pelvic fracture** | **Training Size** | **Validation Size** | **Testing Size** | **Validation Method** |
| --- | --- | --- | --- | --- | --- | --- | --- |
| Cheng, 2021 | 5,204 | 919 | 2,094 | 100% | NR | 1,888 | 5-FCV |
| Chen, 2020 | 2,359 | 759 | 1,600 | 70% | 10% | 20% | 5-FCV |
| Inagaki, 2022 | 2,238 | 770 | 1,468 | 91% | NR | 9% | NR |
| Islam, 2025 | 876 | 472 | 404 | 72% | 18% | 10% | 5-FCV |
| Kassem, 2023 | 876 | 472 | 404 | 70% | 15% | 15% | NR |
| Kitamura, 2020 | 7,337 | 836 | 6,501 | 70% | NR | 30% | NR |
| Lee, 2024 | 940 | 773 | 167 | NR | NR | 100% | 5-FCV |
| Park, 2024 | 1,169 | 972 | 197 | 70% | NR | 30% | NR |
| Rahman, 2024 | 264 X-rays; 6,980 X-rays synthesized from 3D-CT | 3,232 | 4,012 | NR | NR | NR | 5-FCV |
| Rosa, 2023 | 235 | 43 | 192 | NR | NR | 100% | NR |
| Ruitenbeek, 2025 | 1,008 (Pelvis) | 173 | 835 | NR | NR | 100% | NR |
| Tsai, 2025 | 100 | NR | NR | 70% | 15% | 15% | 5 repeated runs |
| Wang, 2025 | NA | NA | NA | 86% | NR | 14% | 5-FCV |
| Wu, 2021 | 1,580 (Pelvis) | NR | NR | NR | NR | 20% | NR |

^a^Abbreviations: NA: Not applicable to the study type; NR: Not reported; 5-FCV: Five-fold cross validation.
